# Assessing Quality of Life Dimensions in Deteriorated Inner Areas: A case from Javadieh Neighborhood in Tehran Metropolis

**DOI:** 10.1007/s11205-015-0986-6

**Published:** 2015-06-12

**Authors:** Samaneh Khaef, Esfandiar Zebardast

**Affiliations:** Urban and Regional Planning, College of Fine Arts, University of Tehran, Enghelab Ave., 14174-66191 Tehran, Islamic Republic of Iran; ITC, Faculty of Geo-Information Science and Earth Observation of the University of Twente, PO Box 217, 7500 AE Enschede, The Netherlands; No 13, Second Street, South Pirouzan Street, Hormozan Avenue, Faze 2, Shahrak e Gharb, 1466634973 Tehran, Islamic Republic of Iran

**Keywords:** Quality of life, Life satisfaction, Deteriorated areas, Physical deterioration indicators

## Abstract

Quality of life is a noticeable concept in urban deteriorated areas where people suffer from multidimensional and complex problems. According to Tehran Renovation Organization (TRO), a deteriorated area is defined just by three physical indicators of fine grain, lack of permeability and lack of durability. But deteriorated areas suffer from other physical as well as socio economic problems which need to be considered in planning processes. Consequently, assessing the QOL in deteriorated inner areas is the main purpose of this paper to survey the overall life satisfaction, to extract main and different aspects of QOL and to determine the extent that overall life satisfaction is explained by different components of life. *Javadieh* neighbourhood, located in Tehran metropolis, one of the most deteriorated neighbourhoods in the city has been chosen as a case for this study. Forty nine indicators which cover different dimensions of quality of life have been selected through literature review. Multi-stage sampling technique for sampling has been applied. In first stage by application of Cochran sampling method, the required sample size has been determined. Then by use of systematic sampling method, questionnaires have been distributed among the residents of the neighbourhood. After data collection, a confirmatory factors analysis indentified 11 factors as identical components of QOL. A stepwise regression is performed to investigate the overall life satisfaction and the extent that QOL is determined by identified domains. Results show that traffic, mobility, housing and infrastructure are the most important aspects of QOL which affect the overall life satisfaction of the residents of the surveyed deteriorated neighbourhood. Also a negative relationship was found between overall satisfaction and private life. The findings of the study also show that the three physical indicators used by the TRO for identifying the deteriorated areas are not adequate to address the deterioration issues. Other physical and socio economic aspects which are incorporated with different QOL dimensions also affect the overall life satisfaction, which have to be addressed in planning and policy making to upgrade quality of life for people in deteriorated neighbourhoods.

## Introduction

Urban deteriorated contexts which have occupied vast areas of Tehran Metropolis need to be considered by planners in planning and policy making processes to provide better life condition for its residents. In fact, considering multidimensional and complex problems within these areas, they have to be recognized in order to provide better plans to upgrade deterioration issues.

Tehran Renovation Organization is the main planning body in city which is responsible for deterioration issues and prepares and implements community renovation and regeneration plans to upgrade the deteriorated areas of the city. Based on the definition provided by the TRO, urban deteriorated areas are defined just by three physical indicators of fine grain, lack of permeability and durability (Tehran Renovation Organization 2010) while other physical, social, environmental, economic and other issues are ignored. Considering the definition of deteriorated areas provided by TRO, there are many criticisms towards these indicators and different renovation experiences done by TRO imply the inappropriateness of the mentioned indicators in identification of deteriorated areas within the city (Haeri 2007).

Nowadays there are many new-built apartments, which have small plot sizes. In addition, many apartments are built which may not be considered as durable by structural engineering standards. Moreover, many parts of Tehran city suffer from lack of proper accessibility. So by adoption of TRO approach all these building types could also be included in the deteriorated area category while they are not. Furthermore, adopted indicators by TRO cover only the physical aspects of QOL and other dominant aspects of life in the deteriorated areas, such as insecurity, unemployment, pollution, lack of affordability are ignored (Kamanroudi 2007). So, these three indicators are not sufficient to define deterioration and many TRO rehabilitation actions by application of these three defined indicators simply show the inappropriateness of TRO approach for addressing the deterioration issues of the city.

Consequently, investigation in aspects of QOL within deteriorated areas from people’s perspective can reveal issues that have to be taken into account in planning processes.

This study specifically aims to:

1. Determine different aspects of QOL within deteriorated areas
2. Survey in people’s overall life satisfaction within deteriorated areas
3. Determine the most important aspects which negatively or positively affect people’s life satisfaction in deteriorated neighbourhoods

This paper is organized as follows: first, literature about QOL, deteriorated areas and their problems have been briefly reviewed. In section of methodology, by application of closed questionnaires in Likert scale, people’s satisfaction towards different QOL aspects have been collected and ranked. Then by use of confirmatory factor analysis and stepwise regression analysis the most important aspects of QOL have been identified and explained in the latter parts of the paper.

### Quality of Life

Quality of life (QOL) which relates to people’s awareness towards their life conditions, has gained much interest in urban studies recently (Nooraie and Tabibian 2012; Eby et al. 2012; Tuan Seik 2000; Ibrahim and Chung 2003). Relevant literatures show that the concept of QOL has been investigated from different fields which implies its multidimensional nature (Eby et al. 2012; Li and Weng 2007; Marans 2003; Mercier et al. 1998; Mulvey 2002; Türksever and Atalik 2001). In fact due to multidisciplinary nature of QOL, it has been investigated more than before from different fields of geography, sociology, environment and economy (Li and Weng 2007; Tuan Seik 2000; Wish 1986).

Importance of QOL originates from lack of understanding people’s real needs and expectation by planners (Khosla 2005). In fact as QOL results can be used and addressed by urban planners and policy makers in resource allocation and development plans (Nooraie and Tabibian 2012; Ibrahim and Chung 2003; Tesfazghi et al. 2010; Türksever and Atalik 2001; Ülengin et al. 2001), it has been considered by planners and policy makers more than before.

In definition of QOL, it has to be noted that it is a broad concept which describes “how well communities support resident well-being and life satisfaction”(Mulvey 2002, p. 656). According to literatures, there is no one single, strict, universally accepted definition for QOL (Apparicio et al. 2008; Das 2008; McCrea et al. 2011; Royuela et al. 2009; Ülengin et al. 2001). Thus, it could be seen that the concepts of liveability and quality of place are sometimes used to define QOL (Li and Weng 2007). Also studies by Rod McCrea et al. (2011) show that “happiness”, “life satisfaction” and “well-being” in several studies have been used to define the concept of QOL.

To investigate the concept of QOL, objective and subjective approaches have been used, which are called objective and perceptual perspectives (Nooraie and Tabibian 2012; Tesfazghi et al. 2010; Royuela et al. 2009; Tuan Seik 2000). Objective approach reflects tangible condition of environment (Das 2008), while subjective measures are referred to individual appraisal of objective condition of their life (Royuela et al. 2009; Das 2008; Malkina-Pykh and Pykh 2008; Shin et al. 2003).

In subjective quality of life approach, level of people’s satisfaction or dissatisfaction with different aspects of life is considered (Royuela et al. 2009), while objective approach measures QOL based on tangible and measurable condition of environment (Royuela et al. 2009; Das 2008).

Considering the two mentioned perspectives in QOL studies, there are different criticisms toward these approaches. According to Lee (2008), QOL study should be assessed in subjective approach and by asking people directly about their life conditions.

In fact as objective QOL may not accurately reflects people’s perception, many researchers such as Nooraie and Tabibian (2012), Eby et al. (2012), Zebardast (2009), Lee (2008), McCrea et al. (2006), Ibrahim and Chung (2003) have applied subjective approach for measuring QOL. They believe that as subjective measures give more valuable information about people’s perception, they are preferred over objective measures (McCrea et al. 2006; Ibrahim and Chung 2003).

As subjective QOL is referred to individual opinion, context plays an important role in their opinion toward their living environment (Marans 2003). In fact people in different contexts by having different conditions, have different concern about different aspects of life.

Consequently overall life satisfaction in different contexts is expressed by different components of life, which have to be recognized to upgrade quality of life for people. Considering importance of context in QOL researches, urban deteriorated area as a noticeable context where people suffer from different aspects that affect their life, has been surveyed in this research.

### Urban Deteriorated Areas

According to literatures, there is no one single and agreed definition for deteriorated areas. Basically depressed, decayed, degeneration, erosion and blighted all refer to urban deteriorated areas where people deal with different issues. In fact, each of these concepts covers different dimensions of deterioration (Tiscali Encyclopaedia 2008). So different definitions for deteriorated areas imply its multidimensional nature and non-physical problems exist in these areas.

However it can be seen that while deterioration covers different dimensions, according to TRO it is recognized by three mere physical indicators of fine grain, lack of permeability, and durability, and other aspects such as environmental, social, transportation and economic aspects are not addressed.

Considering the above-mentioned definition of deterioration, there are many criticisms towards these indicators. Different applied renovation experiences by TRO also show the inadequacy of the mentioned indicators (Haeri 2007).

### QOL Indicators

As discussed earlier, QOL is a multifaceted concept which has been studied from different points of view (Nooraie and Tabibian 2012; Eby et al. 2012; Li and Weng 2007; Marans 2003; Mulvey 2002; Türksever and Atalik 2001). Consequently different studies have applied different indicators to measure QOL and there is no standard method for selection of indicators (Diener 1995).

According to Malkina-Pykh and Pykh (2008) in order to measure QOL; all indicators have to meet the following requirements:

1. Help policy makers and planners to assess and develop their plans
2. Have clear practical purpose
3. Be reliable, valid and sensitive
4. Be potentially neutral
5. Be simple and understandable
6. Be locally relevant

Considering different QOL studies, to select appropriate indicators which best depict QOL condition in the selected study area, relevant literatures have been reviewed. Then based on literature review and considering the local conditions and characteristics, 44indicators from different dimensions of life, are selected for the purposes of this study which are reflected in Table 1.

**Table 1:** Selected indicators to measure QOL in current research

	Indicators	Study
Environment	Quietness	Das (2008), McCrea et al. (2006), Marans (2003)
	Cleanliness	Ülengin et al. (2001), Santos and Martins (2007), Foo (2000),
	Air pollution	Das (2008)
	Environmental health	Lee and Guest (1983)
Social life	Safety	Santos and Martins (2007), Foo (2000), Ülengin et al. (2001), Rahman et al. (2005)
	Intention to stay	Eby et al. (2012)
	Supportive friends and neighbors	Das (2008), McCrea et al. (2006)
Personal relationship	Sufficient money	Becker (2007), Tesfazghi et al. (2010), Das (2008)
	Life expenses	Ülengin et al. (2001), Becker (2007), Tesfazghi et al. (2010), Das (2008)
	Self-energy	Nooraie and Tabibian (2012), WHO (1996)
Housing	Housing facilities	Ülengin et al. (2001), Foo (2000), Das (2008)
	Number of rooms	Ülengin et al. (2001), Foo (2000), Das (2008), Zebardast (2009)
	Ventilation condition	Ülengin et al. (2001), Foo (2000), Das (2008)
	Housing space	Royuela et al. (2009), Tesfazghi et al. (2010), Zebardast (2009)
	Housing infrastructure	Ülengin et al. (2001), Foo (2000), Das (2008)
	House durability	Royuela et al. (2009), Zebardast (2009)
	Privacy in housing	Royuela et al. (2009), Tesfazghi et al. (2010), Zebardast (2009)
Access to educational services	Access to kindergarten	Lee (2008), Foo (2000), Santos and Martins (2007)
	Access to primary school	Lee (2008), Foo (2000), Santos and Martins (2007)
	Access to elementary school	Lee (2008), Foo (2000), Santos and Martins (2007)
	Access to high school	Lee (2008), Foo (2000), Santos and Martins (2007)
Access to daily facilities	Access to official and administrative centers	McCrea et al. (2006), Santos and Martins (2007), Das (2008), Lee (2008)
	Access to shopping centers	McCrea et al. (2006), Santos and Martins (2007), Das (2008), Lee (2008)
	Access to bank	Das (2008), Lee (2008)
	Access to health care centers	Ülengin et al. (2001), Foo (2000), Santos and Martins (2007), McCrea et al. (2006), Das (2008), Marans (2003)
Access to recreational services	Access to park	Ülengin et al. (2001), Foo (2000), Santos and Martins (2007), Das (2008), Marans (2003)
	Access to recreational center	Ülengin et al. (2001), Foo (2000), Ulengin et al. 2001), Lee (2008)
	Access to sport centers	Santos and Martins (2007), McCrea et al. (2006)
	Access to cultural centers	Ülengin et al. (2001), Foo (2000)
Access to transportation services	Access to bus station	Ülengin et al. (2001), Foo (2000)
	Access to minibus station	Ülengin et al. (2001), Foo (2000)
	Access to public taxi	Ülengin et al. 2001), Foo (2000)
	Access to private taxi	Ülengin et al. (2001), Foo (2000)
	Access to metro station	Ülengin et al. (2001), Foo (2000)
Traffic and mobility	Mobility condition	Foo (2000), Das (2008), Lee (2008)
	Public transportation expense	Foo (2000), Ülengin et al. (2001)
	Pedestrian mobility	Ülengin et al. (2001), Foo (2000), Das (2008), Lee (2008)
	Easiness in access to transport facility	Ülengin et al. (2001), Foo (2000), Das (2008), Lee (2008)
	Safety against accident	Marans (2003), Tesfazghi et al. (2010)
Infrastructure	Garbage collection system	Santos and Martins (2007), Lee (2008), Foo (2000), Das (2008), Ülengin et al. (2001)
	Water system	Ülengin et al. (2001), Foo (2000), Santos and Martins (2007), Lee (2008)
	Telephone system	Ülengin et al. (2001), Foo (2000), Santos and Martins (2007), Lee (2008)
	Gas system	Ülengin et al. (2001), Foo (2000), Santos and Martins (2007), Lee (2008)
	Electricity	Ülengin et al. (2001), Foo (2000), Santos and Martins (2007), Lee (2008)

### Study Area

Tehran metropolis located in Tehran province of Iran is consisted of 22 districts with different physical and socio economic characteristics. Based on three mentioned indicators by TRO, 149 hectares of district 16 of Tehran is identified as deteriorated areas where *Javadieh* is one of its neighbourhoods that has the highest deterioration rate. *Javadieh* neighbourhood covers an area of 120.62 hectares.

*Javadieh* neighbourhood is located in north-west of district 16 and suffer from high deterioration and deprivation. This neighbourhood is adjacent to major urban spaces of Velayat Park and the Tehran railway station. However as *Javadieh* is bordered by a major highway, it has been marginalized and this has added to its deprivation and to socio economic and physical problems, which have been investigated in the current study.

According to official census, in 1996 *Javadieh* has been consisted of 52,677 people which has been reduced to 47,780 people in the year 2006. So during the 10 year period between 1996 and 2006, about 4887 people have left the neighbourhood. High deterioration and lack of facilities are main reasons for this population reduction.

**Fig. 1:**
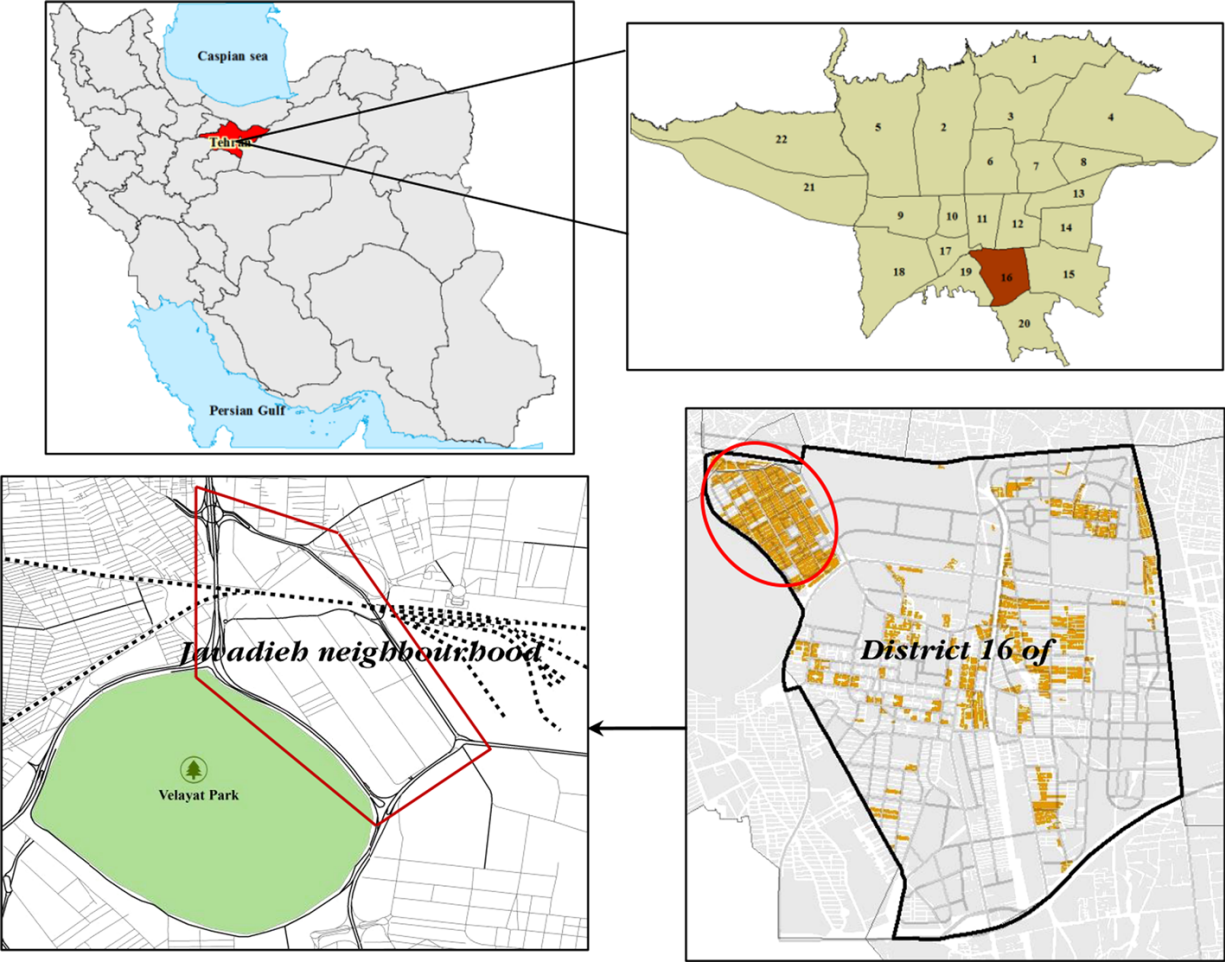
Status of deterioration in Javadieh and districts of 16 of Tehran City. *Source*: Author elaboration based on Tehran municipality of district 16

So considering the objectives of this research, *javadieh* neighbourhood is selected as a case study in this research. Figure 1 shows status of deterioration and location of *Javadieh* in Tehran city.

## Methodology

### Sample Selection

As this research aims to investigate different aspects of QOL in deteriorated areas, selection of a neighbourhood based on administrative boundaries will not meet this research objective. In fact there are significant varieties within each administrative boundary in city which needs to be studied separately. Moreover people in two adjacent administrative boundaries might have similarities in terms of using same services and facilities.

Therefore to identify different aspects of QOL in deteriorated areas, first, three most deteriorated districts in the city were identified: districtes 2, 4, and 16 (according to the TRO definition).Then considering the deterioration rate and also other social and economic aspects as well as aspects of deterioration, district 16 was chosen for the purposes of this study.

District 16 consists of 7 neighbourhoods with different status of deterioration. Table 2 shows the pattern of deteriorated among the 7 neighbourhoods in district 16. This table shows that *Javadieh* has the highest deterioration rate.

**Table 2:** Level of deterioration across the neighborhoods in district 16 of Tehran City

Ranking based on deterioration rate	Deteriorated rate	Neighbourhoods
1	38.87	1 (Javadieh)
2	24.69	2
3	8.51	3
4	64.71	4
5	0.75	5
6	3.72	6
7	69.7	7

*Source:* Author elaboration based on Tehran renovation organization

After selection of *Javadieh* as the case study, the sample size for this neighbourhood has to be identified. So considering the population of this neighbourhood in official survey of 2006 (47,780 people) and applying the Cochran’s sampling formula (1977), with a maximum acceptable error (d = 0.05) and confidence level (z = 1.96) and (p and q = 0.5), 381 households have been identified as the sample size.

### Household Interviews

To avoid ambiguity in questions, to increase number of collected questionnaires and to extract main issues in deteriorated context of *Javadieh*, structured interviews by application of questionnaires were conducted.

First, pilot pre-tests by application of Cronbach’s Alpha as a tool to assess the reliability of applied questions were conducted with 45 households. Cronbach’s Alpha value ranges from 0 to 1. Cronbach’s Alpha for this study is 0.8. Based on to Nunnally (1978), values of 0.7 and over are considered as acceptable reliability coefficients. So the test and applied questions could be considered as reliable.

After testing for reliability of provided questions, in order to investigate the satisfaction from different aspects of life, structured interviews were conducted. Respondents who were household heads or housewives were selected randomly and were interviewed from 1st April till 3th August 2011.

Provided questions have been measured in 5-point Likert scale, where 1 shows total dissatisfaction and 5 shows total satisfaction. Some of questions are as follow: level of satisfaction from; existing park and green spaces, recreational centres, Cleanliness, quietness, metro and bus stations, fire stations, educational centres, cultural centres, groceries and shopping stores, post offices, personal safety, safety and convenience for women and children, housing facilities and etc. Also respondents were asked to reflect their level of intention to stay in their neighbourhood, level of communication with their neighbours, supportive friends and so on.

Moreover to capture different aspects of QOL, which might have not been considered in the questionnaire, at the end respondents were asked to reflect their comments about their neighbourhood. While analysing the open question is difficult, but it was useful to understand what truly interests the respondents.

## Data Analysis

### Factor Analysis

Factor analysis is a multivariate analytical technique which is applied to extract a subset of uncorrelated variables called factors that explain the variance observed in the original dataset (Everitt and Dun 1991). In fact, many indicators are applied in a research that may affect people’s subjective QOL. So to select indicators among all applied indicators that best describe QOL, factor analysis is applied.

There are two types of factor analysis: confirmatory and explanatory analysis. In this research as applied indicators are related to specific categories, confirmatory factor analysis were adopted which categorized indicators in 11 domains as follow: satisfaction from social life, personal relationship, environment, housing, infrastructure, access to recreational service, access to educational service, access to daily facilities, access to transportation service, traffic and mobility and total satisfaction.

Then in order to see suitability of the selected domains for applied indicators in questionnaires, Bartlett’s sphericity test and the Kaiser–Meyer–Olkin (KMO) measure for sampling adequacy were tested. According to Table 3, for all categories, Bartlett’s Sphericity Test and KMO measure indicate suitability of selected domains and their included indicators.

**Table 3:** KMO and Bartlett’s sphericity test for confirmatory factor analysis

Domains	F1	F2	F3	F4	F5	F6	F7	F8	F9	F10	F11
KMO measure of sampling adequacy	0.61	0.62	0.78	0.88	0.82	0.61	0.74	0.69	0.76	0.79	0.79
Bartlett’s sphericity test Sig.	0.000	0.000	0.0001	0.000	0.000	0.000	0.000	0.000	0.000	0.000	0.000

F1, social life; F2, personal relationship; F3, environment; F4, housing; F5, infrastructure; F6, access to recreational services; F7, traffic and mobility; F8, access to daily facilities; F9, access to transportation services; F10, access to educational services; F11, total satisfaction

Table 3 shows Eigent values for selected domains and indicators. According to this table, the selected domains significantly reflect subjective QOL and consequently all 11 domains are used for further analysis.

In order to determine the number of factors in each 11 domains of QOL, Kaiser Criterion (Kaiser 1960) was used. Based on this criterion, factors with eigenvalues greater than or equal to 1 are accepted as possible sources of variance in the dataset, with the highest priority ascribed to the factor that has the highest eigenvector sum (Zebardast 2009).

According to Table 4 it can be seen that all associated indicators are highly correlated with their selected domains and as a result mentioned domains properly reflect their indicators. As a result, selected indicators in 11 domains are suitable for reflection of QOL components.

**Table 4:** Factor loading matrix for indicators and adopted domains

Access to educational service		Access to daily service		Access to recreational service	
Indicators	Factor loading	Indicators	Factor loading	Indicators	Factor loading
Access to kindergarten	0.71	Access to official centers	0.60	Access to park	0.71
Access to primary school	0.83	Access to shopping centers	0.75	Access to recreational center	0.93
Access to elementary school	0.89	Access to bank	0.74	Access to sport centers	0.92
Access to high school	0.89	Access to hospital	0.72	Access to cultural centers	0.42
% Explained variance	69.86	% Explained variance	49.958	% Explained variance	59.77

Access to transportation facilities		Traffic and mobility		Infrastructure	
Indicators	Factor loading	Indicators	Factor loading	Indicators	Factor loading
Access to bus station	0.59	Mobility condition	0.63	Garbage collection system	0.59
Access to minibus station	0.77	Public transportation expense	0.67	Water system	0.74
Access to public taxi	0.75	Pedestrian mobility	0.63	Telephone system	0.78
Access to private taxi	0.76	Easiness in access to transport facility	0.77	Gas pipe system	0.83
Access to metro station	0.59	Safety against accident	0.55	Electricity	0.79
% Explained variance	48.68	% Explained variance	42.74	% Explained variance	56.53

Environment		Social life		Private relation	
Indicators	Factor loading	Indicators	Factor loading	Indicators	Factor loading
Cleanliness	0.80	Personal safety	0.070	Life expenses	0.83
Air pollution	0.76	Intention to stay	0.77	Self-energy	0.67
Environmental health	0.84	Supportive friends	0.65	Sufficient money to handle life	0.83
% Explained variance	61.08	% Explained variance	49.79	% Explained variance	61.31

Housing		Total satisfaction	
Indicators	Factor loading	Indicators	Factor loading
Housing facilities	0.64	Access to facilities	0.68
Number of rooms	0.76	Infrastructure	0.77
Ventilation condition	0.77	Transportation and mobility	0.73
Housing space	0.83	Social life	0.59
Housing infrastructure	0.85	Environment	0.89
House durability	0.79	Housing	0.83
Privacy in housing	0.71		
% Explained variance	59.01	% Explained variance	56.92

### Life Satisfaction

After extraction of QOL components, in order to see the extent that each component affects respondents’ life satisfaction and also component level of importance, stepwise regression analysis was conducted. Total life satisfaction is considered as dependent variable and the extracted components of QOL are considered as predicators: access to educational service (C1), access to daily services (C2), access to recreational services (C3), access to transportation facilities (C4), traffic and mobility (C5), infrastructure (C6), environment (C7), social life (C8), housing (C9) and private life (C10). The result of the stepwise regression analysis is shown in Table 5.

**Table 5:** Stepwise regression analysis results

Factors	Unstandardized coefficients		Standardized coefficients	t	Sig.
	B	Std. error	Beta		
(Constant)	0.006	0.036		0.161	0.872
C5: traffic and mobility	0.237	0.051	0.237	4.671	0.000
C9: housing	0.292	0.044	0.291	6.693	0.000
C6: infrastructure	0.269	0.043	0.268	6.245	0.000
C10: private life	−0.250	0.043	–0.249	−5.861	0.000
C3: access to recreational service	0.179	0.040	0.180	4.504	0.000
C2: access to daily service	0.128	0.043	0.125	2.980	0.003
C7: environment	0.124	0.046	0.125	2.691	0.007
R^2^	0.548				
Adjusted R^2^	0.539				

Based on results presented in Table 5, regression equation is reflected as below:

$$\begin{aligned} Life\,satisfaction & = 0.006 + 0.237C5 + 0.29C9 + 0.269C6 - 0.249C10 + 0.18C3 + 0.125C2 + 0.125C7 \\ R^{2} & = 0.548 \\ \end{aligned}$$

The above equation shows that there is no statistically significant relationship between life satisfaction and access to transportation facility (C4), educational services (C1) and social life (C8). In the regression analysis, beta coefficient refers to the degree of importance of each component. The beta coefficient in Table 5 indicates that “traffic and mobility (C5)”, “housing (C9)”, and “infrastructure (C6)” are the three most important aspects of QOL that significantly affect total life satisfaction in the surveyed neighborhood. The negative beta sign for the private life component (C10) could be anticipated since the private life component is composed of three indicators that explain household’s handling of their life expenditures. Since the neighborhood is predominantly occupied by low-income households, therefore their life satisfaction is adversely affected by their expenditure pattern.

## Conclusion

To survey the different aspects of QOL, based on literatures and deterioration issues, 49 indicators depicting the social, economic, environmental, housing, physical and infrastructure aspects of life were selected and then people’s level of satisfaction towards these selected indicators were questioned. To analyse collected questionnaires, confirmatory factor analysis has been applied. The results show that all indicators could be categorized in 11 domains of: Access to daily services, access to educational services, access to recreational services, access to transportation facilities, traffic and mobility, infrastructure, environment, social life, private relation, housing and total satisfaction.

To investigate the suitability of extracted factors and their indicators, KMO statistics and Barttlet test have been applied. The obtained results reflect the suitability of selected factors and their indicators.

Of the 11 identified components of QOL, “traffic and mobility”, “housing” and “infrastructure” are considered as the most important domains of QOL which explained about 42.74, 59.01 and 56.53 % of variance in QOL, respectively. Moreover based on regression analysis, no statistically significant relationship was found between life satisfaction and domains of “access to transportation facilities”, “educational services” and “social life”.

Considering the definition of deterioration applied by the Tehran Renovation Organization from one hand and main aspects of QOL identified in context of *Javadieh* on the other hand, it could be seen that deteriorated issues in context of *Javadieh,* as one of the most deteriorated neighbourhoods”, are not limited to the three TRO indicators. In fact, while TRO deals with all deteriorated neighbourhoods problems by just three aforementioned indicators, daily facility, traffic, mobility, infrastructure and housing are the most important aspects of QOL from people’s perspective, which significantly affect their total life satisfaction.

So while the results of this study show that deterioration issues in studied neighbourhood are mainly physical, but they are not limited to TRO indicators. In fact QOL is a contextual concept and due to different dominant issues in each neighbourhood, deterioration aspects have to be surveyed in each neighbourhood separately and using general indicators may not be applicable to the whole neighbourhood.

Speaking to the contextual situation of *Javadieh* neighbourhood which has been discussed earlier, because of geographical situation of *Javadieh* which is adjacent to major streets, it has been marginalized and consequently traffic and mobility are the main people’s concerns. Moreover as *Javadieh* suffers from high deterioration, lack of sufficient infrastructure and deteriorated houses are other components which have been reflected by the people.

To sum up, speaking to results found in *Javadieh,* to see what matters to people in term of QOL and to improve the deteriorated neighbourhoods, people’s perspectives towards different aspects of life have to be investigated in each neighbourhood separately. As QOL, in contextual concept, application of general indicators for all neighbourhoods may not end to improve the people’s satisfaction towards their lives.
